# PAC1R agonist maxadilan enhances hADSC viability and neural differentiation potential

**DOI:** 10.1111/jcmm.12772

**Published:** 2016-01-22

**Authors:** Xiaoling Guo, Rongjie Yu, Ying Xu, Ruiling Lian, Yankun Yu, Zekai Cui, Qingshan Ji, Junhe Chen, Zhijie Li, Hongwei Liu, Jiansu Chen

**Affiliations:** ^1^ Key Laboratory for Regenerative MedicineMinistry of EducationJinan UniversityGuangzhouChina; ^2^ Department of Cell BiologyJinan UniversityGuangzhouChina; ^3^ GHM Institute of CNS RegenerationJinan UniversityGuangzhouChina; ^4^ Department of OphthalmologyThe First Clinical Medical College of Jinan UniversityGuangzhouChina; ^5^ Department of OphthalmologyAffiliated Anhui Provincial Hospital of Anhui Medical UniversityHefeiChina; ^6^ Department of MathematicsSouth China University of TechnologyGuangzhouChina; ^7^ Eye InstituteMedical College of Jinan UniversityGuangzhouChina

**Keywords:** hADSCs, maxadilan, apoptotic, proliferation, neural differentiation

## Abstract

Pituitary adenylate cyclase‐activating polypeptide (PACAP) is a structurally endogenous peptide with many biological roles. However, little is known about its presence or effects in human adipose‐derived stem cells (hADSCs). In this study, the expression of PACAP type I receptor (PAC1R) was first confirmed in hADSCs. Maxadilan, a specific agonist of PAC1R, could increase hADSC proliferation as determined by Cell Counting Kit‐8 and cell cycle analysis and promote migration as shown in wound‐healing assays. Maxadilan also showed anti‐apoptotic activity in hADSCs against serum withdrawal‐induced apoptosis based on Annexin V/propidium iodide analysis and mitochondrial membrane potential assays. The anti‐apoptotic effects of maxadilan correlated with the down‐regulation of Cleaved Caspase 3 and Caspase 9 as well as up‐regulation of Bcl‐2. The chemical neural differentiation potential could be enhanced by maxadilan as indicated through quantitative PCR, Western blot and cell morphology analysis. Moreover, cytokine neural redifferentiation of hADSCs treated with maxadilan acquired stronger neuron‐like functions with higher voltage‐dependent tetrodotoxin‐sensitive sodium currents, higher outward potassium currents and partial electrical impulses as determined using whole‐cell patch clamp recordings. Maxadilan up‐regulated the Wnt/β‐catenin signalling pathway associated with dimer‐dependent activity of PAC1R, promoting cell viability that was inhibited by XAV939, and it also activated the protein kinase A (PKA) signalling pathway associated with ligand‐dependent activity of PAC1R, enhancing cell viability and neural differentiation potential that was inhibited by H‐89. In summary, these results demonstrated that PAC1R is present in hADSCs, and maxadilan could enhance hADSC viability and neural differentiation potential in neural differentiation medium.

## Introduction

Mesenchymal stem cells (MSCs) can differentiate into lineages for many cell types in the appropriate microenvironment 1, 2, 3, 4. This potential makes MSCs an attractive stem cell source for cell‐based therapies in regenerative medicine and tissue engineering 5, 6, 7. Human adipose‐derived stem cells (hADSCs) are very similar to MSCs in terms of surface antigens 8, 9, and they also possess multipotentiality 10, 11, 12. As adipose tissue can be more easily obtained than other tissues, adipose is a more reliable stem cell source for therapy 13, 14. However, with large‐scale growth procedures required for clinical purposes, it has been revealed that hADSCs often have defective cell viability and apoptosis, which are obstacles for stem cell therapy 15.

PAC1R is a specific receptor of pituitary adenylate cyclase‐activating polypeptide (PACAP), which belongs to the class B G protein‐coupled receptor family 16. PAC1R is primarily located in the central nervous system, peripheral nervous system, lungs and respiratory neural tissues 17, 18. PAC1R also mediates a variety of biological functions such as nerve injury repair 19 and the regulation of vascular 20 and metabolic balance 21. PAC1R is involved in cell viability and differentiation. For example, studies have reported that activated PAC1R can protect certain types of cells against apoptosis, such as the olfactory epithelium and olfactory placodal cells 22, cochlear cells 23, cardiomyocytes 24 and cortical neurons 25. Recent data has shown that PAC1R has dimer‐dependent activity that promotes cell proliferation and inhibits apoptosis through the Wnt/β‐catenin signalling pathway 26. Nielsen *et al*. found that PAC1R was expressed during the peak period of neuronal differentiation in nascent dorsal root ganglions, and blockade of the PAC1R receptor inhibited neuronal differentiation 27.

Maxadilan, isolated from the salivary glands of the sand fly Lutzomyia longipalpis, is a 61‐amino acid vasodilatory peptide 28. Although it has no prominent sequence homology with PACAP, maxadilan is a PAC1R‐specific agonist and is used to assay the diverse physiological functions of PAC1R 29, 30, 31. For example, maxadilan is used to determine that PAC1R activation prevents TNF‐mediated cell death in olfactory placode cells 22.

In this study, PAC1R expression in hADSCs was firstly investigated, and maxadilan was subsequently used to probe the anti‐apoptotic effect of PAC1R in hADSCs. This study also attempted to elucidate whether maxadilan facilitates hADSC growth or enhances hADSC neural differentiation *in vitro*. To evaluate the effects of maxadilan on neural differentiation potential in hADSCs, we quantitatively examined induced hADSC morphology in terms of cell sizes, protrusion numbers and protrusion lengths using a custom‐designed program written in MATLAB (version 7.11.0; MathWorks, Natick, MA, USA). Furthermore, the signalling pathways mediated by PAC1R were simply detected to confirm the effects of maxadilan on hADSCs. These pathways included the Wnt/β‐catenin signalling pathway, which is associated with PAC1R dimer‐dependent activity, and the PKA signalling pathway, which is associated with PAC1R ligand‐dependent activity and are involved in cellular proliferation, anti‐apoptosis or neural differentiation.

## Materials and methods

### hADSC isolation and culture

Human adipose tissues were obtained from the abdomen and the thigh of five healthy female donors with a mean age of 31 ± 5 years through liposuction after written informed consent was obtained. The institutional ethical review board of the First Affiliated Hospital of Jinan University approved the protocols. Methods for securing human tissue were in compliance with the Declaration of Helsinki. The adipose tissues were microdissected under a stereoscope (1 mm^3^) and then washed five times to remove blood clots, red blood cells and local anesthetics with sterile PBS. The remaining adipose tissues were incubated with Low‐Glucose DMEM (LG‐DMEM) containing 0.1% type I collagenase (Sigma‐Aldrich, Louis, MO, USA) for 1 hr at 37°C on a shaking platform. The digested adipose tissues were filtered with a 40 μm cell strainer and then centrifuged (300 × g, 10 min.). The supernatant was discarded and deposit was suspended in LG‐DMEM containing 100 U/ml penicillin/streptomycin (P/S) and 10% foetal bovine serum (FBS; Gibco, Grand Island, NY, USA). Last, the cell suspension was seeded into 25 cm^2^ Petri dishes and incubated in a 37°C incubator with 5% CO_2_. The hADSC culture medium (LG‐DMEM, 100 U/ml P/S, 10% FBS) was changed every other day.

### Flow cytometry assays for cell surface antigen

Flow cytometry assay was performed to detect the immunophenotypes of hADSCs as a previous report 32. Briefly, hADSCs at passage 1 (P1) were harvested and washed with PBS. Then, cells were incubated with phycoerythrin‐conjugated or fluorescein isothiocyanate (FITC)‐conjugated monoclonal antibodies: CD44, CD59, CD29, CD105, CD34, CD45 and HLA‐DR (BD, San Diego, CA, USA) in the dark at 4°C for 20 min. Then, cells were washed with PBS and centrifuged (400 × g, 5 min.), and suspended in 200 μl of PBS. CD surface antigens were analysed with Flow Cytometer (BD FACSAria, San Diego, CA, USA).

### Adipogenic and osteogenic differentiation *in vitro*

Adipogenic induction of hADSCs followed a previous reported procedure 33. Human adipose‐derived stem cells at P2 were induced in an adipogenic induction medium (Sigma‐Aldrich) for 12 days. Then, the cells were fixed with 10% paraformaldehyde for 30 min. and washed three times with PBS. The cells were stained with 2% fresh Oil red‐O solutions (Sigma‐Aldrich) for 10 min. The cells were washed with 70% alcohol and were imaged using an inverted microscope (OLYPUS, Tokyo, Japan).

Osteogenic induction of hADSCs was performed according to a previously reported method 34. hADSCs at P2 were induced in an osteogenic induction medium (Sigma‐Aldrich) for 12 days. Then, the cells were fixed with 4% paraformaldehyde for 10 min. and washed three times with PBS. The cells were stained with 10 mg/ml alizarin red (Sigma‐Aldrich) for 10 min. The cells were gently washed three times with deionized water and imaged with an inverted microscope.

### PCR assays

Total RNA was extracted from cells using a Tissue RNA Miniprep Kit (Invitrogen, Hudson, CA, USA), and the concentration of RNA was quantified by measuring OD at 260 nm. Soon RNA (1 μg) was reverse transcribed in a 10 μl reaction mixture containing 0.5 μl RT Enzyme Mix, 0.5 μl Primer Mix, 2 μl 5× RT Buffer, 2–6 μl nuclease‐free water at 37°C for 1 hr and at 98°C for 5 min. The cDNA synthesis was performed with a cDNA Synthesis Kit (TOYOBO, Tokyo, Japan), and the expression of PAC1R and GADPH was assessed by RT‐PCR. The PCR mixture was first denatured at 94°C for 2 min. and then amplified for 35 cycles (94°C, 30 sec.; 60°C, 30 sec.; 72°C, 30 sec.) using an authorized thermal cycler (Eppendorf, Hamburg, Germany).

The expression levels of Nestin, MAP‐2, NF‐M, neuron‐specific enolase (NSE) and Tuj‐III were analysed by qPCR using a SYBR Green PCR Kit (Takara, Otsu, Japan). The reaction mixture consisted of 10 μl SYBR Green Mix, 0.8 μl forward, 0.8 μl reverse primers, 1 μg diluted cDNA and 5–8 μl ddH_2_O. The reaction process was as follows: 95°C for 3 min., followed by 40 cycles of 95°C for 10 sec. and 59°C for 30 sec. The relative expression of genes was normalized to GAPDH. The melting curve was examined for the quality of PCR amplification for each sample, and quantification was performed with the comparative 2^−ΔΔCt^ method. The primer sequences were shown in Table 1.

**Table 1:** List of primers

Primers	Sequences (5′–3′)	Product length	GeneBank number
PAC1‐F	TGGCATTATCGTCATCCTTG	310 bp	NM_001118.4
PAC1‐R	GAAGTCCACAGCGAAGTAACG	310 bp	NM_001118.4
GADPH‐F	CCACTAGGCGCTCACTGTTC	180 bp	NM_001289746.1
GADPH‐R	TTGAGGTCAATGAAGGGGTCA	180 bp	NM_001289746.1
MAP‐2‐F	GCTGCATATGCGCTGATTCT	255 bp	NM_002374.3
MAP‐2‐R	AGATGCCTCTGTTAGCGGTG	255 bp	NM_002374.3
NF‐M‐F	GCCGCAGACCTAGGGTATTT	174 bp	NM_001105541.1
NF‐M‐R	ACCGAATTCATCCCTCCTGC	174 bp	NM_001105541.1
NSE‐F	TCCCGAGATCCCAGCCATCA	183 bp	NM_001975.2
NSE‐R	TGTTTGTCTCCATCCCTCAGC	183 bp	NM_001975.2
Tuj‐III‐F	ACACCATGCACCAAGACCAA	163 bp	NM_001123066.3
Tuj‐III‐R	CCCTCATCCACTAAGGGTGC	163 bp	NM_001123066.3
Nestin‐F	AACAGCGACGGAGGTCTCTA	220 bp	NM_006617.1
Nestin‐R	TTCTCTTGTCCCGCAGACTT	220 bp	NM_006617.1

### Western blot assays

Cells were washed with PBS and then lysed in radio‐immunoprecipitation assay (RIPA) lysis buffer containing protease inhibitor PMSF (Bocai Biotechnology, Shanghai, China) to obtain total proteins. The protein concentration of samples was measured with BCA^™^ Protein Assay Kit (Takara). A total of 50 μg of proteins were then separated by 12% SDS‐PAGE and transferred to polyvinylidene fluoride membranes. The membranes were blocked with 5% non‐fat dry milk (Cell Signaling, Danfoss, MA, USA) in tris‐buffered saline tween‐20 (TBST) buffer for 1 hr. Then, the membranes were incubated overnight at 4°C with primary antibodies as follow: rabbit polyclonal anti‐PAC1R antibody (1:3000; Santa Cruz Biotechnology, Santa Cruz, CA, USA), rabbit polyclonal anti‐Cleaved Caspase 3 (1:1000; Santa Cruz Biotechnology), rabbit polyclonal anti‐Cleaved Caspase 9 (1:1000; Santa Cruz Biotechnology), rabbit polyclonal anti‐MAP‐2 (1:5000; Sigma‐Aldrich), mouse polyclonal anti‐NF‐M (1:5000; Abcam, Cambridge, MA, USA), chicken polyclonal anti‐NSE (1:5000; Merck Millipore, Billerica, MA, USA), mouse monoclonal anti‐Nestin (1:3000; Santa Cruz Biotechnology), rabbit polyclonal anti‐Tuj‐III (1:3000; Abcam), rabbit polyclonal‐β‐catenin (1:5000; Santa Cruz Biotechnology), rabbit polyclonal‐Cyclin D1 (1:3000; Santa Cruz Biotechnology), rabbit polyclonal‐c‐myc (1:3000; Santa Cruz Biotechnology), rabbit polyclonal anti‐survivin (1:3000; Santa Cruz Biotechnology), mouse monoclonal anti‐β‐actin (1:1000; Santa Cruz Biotechnology) and rabbit polyclonal anti‐GADPH (1:10,000; Bioworld, Minneapolis, MN, USA). Then, the membranes were incubated with HRP‐conjugated antimouse, anti‐chicken or anti‐rabbit IgG secondary antibodies (1:10,000; Bioword) for 1 hr at room temperature and washed three times with TBST. After adding enhanced chemiluminescence detection regents (Pierce, Rockford, IL, USA), the membranes were visualized by scanning the immunostaining band (Tanon2500, Tanon Science & Technology Inc., Shanghai, China). The intensity of band was analysed with ImageJ software (National Institutes of Health Inc., Bethesda, MD, USA).

### Immunofluorescence assays

Immunofluorescence assays were conducted as a previous description 35. Briefly, after fixation using 4% paraformaldehyde for 15 min. at room temperature, cells were washed three times with PBS. Then, cells were permeabilized with 0.1% TritonX‐100 for 15 min., and incubated with 3% (w/v) bovine serum albumin for 60 min. at room temperature. Then, cells were incubated with mouse monoclonal anti‐Vimentin (1:500; Boster, Guangzhou, China), rabbit polyclonal anti‐MAP‐2 (1:1000; Sigma‐Aldrich), mouse polyclonal anti‐NF‐M (1:1000; Abcam), rabbit polyclonal anti‐Tuj‐III (1:1000; Abcam), chicken polyclonal anti‐NSE (1:1000; Merck Millipore) and mouse monoclonal anti‐Nestin (1:500; Santa Cruz Biotechnology) for a night at 4°C, and then with Cy3‐conjugated antimouse, FITC‐conjugated anti‐rabbit, FITC‐conjugated anti‐chicken IgG secondary antibodies (1:5000; Bioword) for 60 min. at room temperature. Then, cells were rinsed with PBS thrice for 5 min. each time. Following, samples were incubated for 15 min. with O,O‐dimethyl‐o‐2,2‐dichlorovinyl phosphate (DAPI) and washed three times with PBS. Last, samples were examined by an inverted microscope (OLYPAS).

### Cell Counting Kit‐8 assays

Cell Counting Kit‐8 (CCK‐8; KeyGEN, Nanjing, China) was used to assay the growth kinetics of hADSCs. Briefly, hADSCs were harvested and seeded in 96‐well plate (1 × 10^4^ cells/well). The next day, cells were washed with PBS and cultured in hADSC culture medium containing maxadilan at 37°C for 24 hrs. Maxadilan was the gift from Dr. Rongjie Yu. It is a recombinant protein prepared using intein mediated rapid purification system, which has been published in a previous report 36. The maxadilan concentration used here contained 0, 20, 40, 60, 80, 100, 120 and 200 nM. Then, each well was added 10 μl CCK‐8 solutions at 37°C for 3 hrs. Finally, the absorbance at 450 nm was immediately measured with a microplate reader. Data analysis was conducted using GraphPad Prism 5 software (GraphPad Software Inc., La Jolla, CA, USA).

### Cell cycle assays

The cell cycle distribution was analysed by flow cytometry with minor modifications 37. After treatment with or without 80 nM maxadilan for 2 days, hADSCs were harvested and fixed with pre‐cooled 70% ethanol at −20°C overnight. Then, fixed cells were washed with PBS and stained with propidium iodide (PI; Sigma‐Aldrich) for 15 min. at room temperature. The stained cells were detected using a Flow Cytometer and data were analysed using MultiCycle software (BD FACSAria Inc., San Diego, CA, USA).

### Scratch wound‐healing assays

Scratch wound‐healing assays were performed as a previous report with minor modifications 38. A confluent monolayer of hADSCs at P2 was mechanically wounded with a 1 ml plastic pipette tip to create a uniform wound in cells grown in 6‐well plates. Then, cells were washed with PBS and cultured in hADSC medium with or without 80 nM maxadilan. Finally, wound closures were recorded at 12 and 24 hrs with phase‐contrast microscopy. The wound areas were calculated using Image J software.

### Annexin V and PI assays

Human adipose‐derived stem cells (1 × 10^6^ cells/well) at P2 were cultured in hADSC medium with or without 80 nM maxadilan for 48 hrs. Then, cells were exposed to serum withdrawal to induce apoptosis for another 24 hrs. Human adipose‐derived stem cells in control were cultured in medium without 80 nM maxadilan and serum withdrawal treatments. Cells were harvested and washed with cold PBS, and then resuspended in 200 μl of Annexin V‐binding buffer. After stained with 2 μl of PI and 2 μl of FITC‐labelled Annexin V (Sigma‐Aldrich), cells were immediately analysed using Flow Cytometer.

### Mitochondrial membrane potential assays

The mitochondrial membrane potential (▵Ψ m) was analysed as a previous document with minor modifications 39. Human adipose‐derived stem cells (1 × 10^6^ cells/well) at P2 were cultured in hADSC medium with or without 80 nM maxadilan for 48 hrs. Then, cells were exposed to serum withdrawal to induce apoptosis for another 24 hrs. Human adipose‐derived stem cells in control were cultured in medium without 80 nM maxadilan and serum withdrawal treatments. Cells were harvested and washed with cold PBS, and then resuspended in 200 μl of tetrechloro‐tetraethylbenzimidazol carbocyanine iodide (JC‐1; Sigma‐Aldrich). After incubation in the dark for 15 min. at room temperature, the cells were immediately analysed using Flow Cytometer.

### Caspase 3 activity assays

A Caspase 3 activity assay kit (Beyotime Bio‐Technologic, Guangzhou, China) was used to detect Caspase 3 activation. Human adipose‐derived stem cells (1 × 10^6^ cells/well) at P2 were cultured in hADSC medium with or without 80 nM maxadilan for 48 hrs. Then, cells were exposed to serum withdrawal to induce apoptosis for another 24 hrs with or without the inhibitors H‐89 (100 μM; Sigma‐Aldrich) or XAV939 (10 μM; Sigma‐Aldrich). Samples were collected and processed according to the manufacturer's instructions. Finally, the absorbance at 405 nm was measured with a microplate reader. Data analysis was conducted using GraphPad Prism 5 software.

### ELISA

The levels of Bcl‐2 in hADSCs administered different treatments were detected with a commercially available ELISA kit (Beyotime Bio‐Technologic) according to the manufacturers' protocols. Assay results were measured with a microplate reader at 450 nm as the test wavelength and 570 nm as the reference wavelength. Data analysis was conducted using GraphPad Prism 5 software.

### The quantification of cellular morphology

The quantification of cellular morphological features from fluorescent images was performed with MATLAB software 40, 41. Briefly, individual cell was segmented in images, and the spreading areas were measured. To quantify the morphologies of protrusions, we extracted the morphological skeletons from individual cell and identified the main body region of the cell with MATLAB software. The protrusions were beyond the main body region and were classified into two types: 1st order protrusions and 2nd order protrusions. The 1st order protrusions directly extended from the cell body, while 2nd order protrusions were those branching from the primary protrusions. The total number of protrusions was determined by the summation of 1st order and 2nd order protrusions. The morphological aspects of cells, including total cell areas, mean cell area, mean length of protrusions, total number of protrusions, total number of 1st order protrusions and total number of 2nd order protrusions, were evaluated based on the measurements from ~60 cells.

### Electrophysiology assays

Cells grown on the coverslips were placed in a recording chamber (1 cm^3^). Voltage‐dependent ionic currents and resting membrane potentials were recorded using the whole‐cell patch clamp technique 42. Patch micropipettes were pulled with an electrode puller (P‐97; SUTTER, San Francisco, CA, USA). Pipettes were filled with the intracellular‐like solutions containing 130 mM CsCl, 20 mM TEA‐Cl, 0.24 mM CaCl_2_, 10 mM N‐2‐hydroxyethylpiperazine‐N‐ethane‐sulphonicacid (HEPES), 10 mM glucose, 5 mM glycol‐bis‐(2‐aminoethylether)‐N,N,N′,N′‐tetraacetic acid (EGTA) and 2 mM Mg‐ATP for recording the inward Na^+^ currents, and pH was adjusted to 7.3 with CsOH (all from Sigma‐Aldrich). For recording the outward K^+^ currents, the pipette solutions contained 120 mM KCl, 5 mM EGTA, 0.24 mM CaCl_2_, 30 mM glucose, 10 mM HEPES and 2 mM Mg‐ATP, and pH was adjusted to 7.3 with KOH (all from Sigma‐Aldrich). The cells were perfused at the rate of 2 ml/min. with standard external solutions comprised of 120 mM NaCl, 3 mM KCl, 2 mM CaCl_2_, 2 mM MgCl_2_, 20 mM glucose and 10 mM HEPES (all from Sigma‐Aldrich). The pH was adjusted to 7.3 with NaOH. Depending upon the type of experiment, 4‐aminopyridine (4‐AP, 2 mM), Cd^2+^ (0.2 mM) and tetrodotoxin (TTX, 1 μM; all from Sigma‐Aldrich) were added into the extracellular medium (4‐AP, Cd^2+^ for recording Na^+^ currents and TTX for recording K^+^ currents). Cells were perfused at 22–24°C, and whole‐cell currents were recorded using an EPC‐10 patch clamp amplifier (HEKA, Lambrecht, Germany). Seal resistance was always greater than 2 GΩ. Membrane currents were low‐pass filtered at 2.9 kHz and sampled at 50 kHz. Current traces were digitized at 0.5 kHz for voltage‐clamp recordings and at 20 kHz for current‐clamp recordings. Cells were held at −80 mV after correcting liquid junction potential (~15 mV) and depolarized from −80 to +40 mV in 5 mV steps. Data recording and measurement were achieved with PatchMaster acquisition software. The peak current under each holding voltage was measured by Igor software, and the I‐V curves were plotted with GraphPad Prism 5.0 software.

### Neural differentiation, dedifferentiation and redifferentiation of hADSCs

Neural induction of hADSCs was performed as a previously report 43. Briefly, hADSCs at P2 were induced in neural induction medium as follows. Chemical neural induction medium, namely DMEM‐F12 supplemented with 2% dimethyl sulphoxide (DMSO), 25 mM KCl, 5 mg/ml insulin, 10 mM forskolin, 1 mM hydrocortisone, 2 mM valproic acid and 200 mM butylated hydroxyanisole (all from Sigma‐Aldrich) for 1 or 3 days.

Cytokine neural induction medium *via* neurotrophic factors, namely DMEM‐F12 supplemented with 50 ng/ml brain‐derived neurotrophic factor (BDNF), 2 mM L‐glutamine, 2% N_2_, 2% B_27_, 1× NEAA (Gibc, Grand Island, NY, USA), 20 ng/ml EGF, and 100 ng/ml bFGF (all from Sigma‐Aldrich) for a week, and then DMEM‐F12 supplemented with 50 ng/ml BDNF, 2 mM L‐glutamine, 20 ng/ml EGF, 2% N_2_, 2% B_27_, 1× NEAA, and 10 μM forskolin (all from Sigma‐Aldrich) for another week.

Human adipose‐derived stem cells first were induced in chemical differentiation medium for 3 days (neural differentiation). Next, the medium was changed out for hADSC culture medium, and the cells were cultured for another 3 days (dedifferentiation). Finally, the medium was changed back to cytokine differentiation medium for 2 weeks (redifferentiation).

### Groups

**Figure 1:**
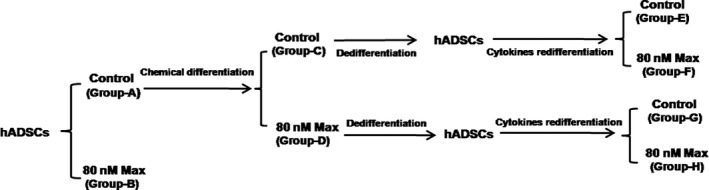
The diagram for grouping in hADSCs with different treatments.

The experimental groups were as follows. Human adipose‐derived stem cells cultured in hADSC medium were used as group‐A and supplemented with 80 nM maxadilan as group‐B. Human adipose‐derived stem cells induced in chemical neural induction medium were used as group‐C and supplemented with 80 nM maxadilan as group‐D. Dedifferentiated and redifferentiated hADSCs in cytokine neural induction medium based on group‐C were used as group‐E and supplemented with 80 nM maxadilan as group‐F. Dedifferentiated and redifferentiated hADSCs in cytokine neural induction medium based on group‐D were used as group‐G and supplemented with 80 nM maxadilan as group‐H. The diagram for grouping was shown in Figure 1.

### Statistical analyses

All data are presented as the mean ± S.E.M. of at least three separate experiments. Statistical significance was evaluated using one‐way anova followed by Dunnett's multiple comparison test. The unpaired Student's *t*‐test was used to compare two different groups. *P*‐value <0.05 was considered to be statistically significant.

## Results

### The identification of hADSCs and PAC1R expression in hADSCs

**Figure 2:**
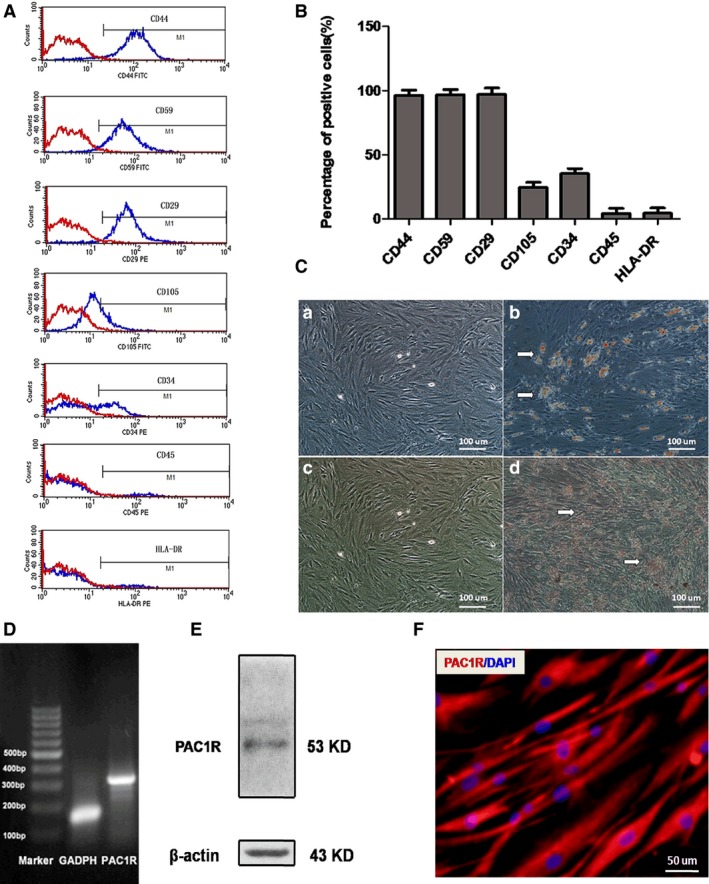
hADSC identification and PAC1R expression in hADSCs. (**A**) Flow cytometry analysis of surface phenotypes of hADSCs. (**B**) The quantitative results of the flow cytometry analysis. (**C**) The adipogenic and osteogenic differentiation of hADSCs. (**a**) Negative control and (**b**) positive group for adipogenic differentiation. (**c**) Negative control and (**d**) positive group for osteogenic differentiation. (**D**) PAC1R expression in hADSCs based on RT‐PCR analysis. (**E**) PAC1R expression in hADSCs based on Western blot assay. (**F**) PAC1R expression in hADSCs based on immunofluorescence assays.

Human adipose‐derived stem cells displayed fibroblastic and spindle‐shaped morphologies. Cells could reach 100% confluence after 5 days of culture. Subsequently, cells were passaged using 0.25% EDTA‐trypsin. The cell surface phenotypes of hADSCs at P1 were assayed using flow cytometry (Fig. 2A). The results showed that hADSCs positively expressed high levels of the cell markers CD44, CD59 and CD29, low levels of CD105 and CD34 and barely expressed CD45 and HLA‐DR (Fig. 2B). In addition, hADSCs at P2 could successfully differentiate into osteogenic and adipogenic lineages (Fig. 2C). Human adipose‐derived stem cells were positively stained by Oil red‐O solution after adipogenic induction on day 12 (Fig. 2 Cb). Extracellular matrix (ECM) calcification was positively stained by alizarin red after osteogenic induction on day 12 (Fig. 2Cd). The specific indicators of osteogenic differentiation were the secretion of ECM with rich collagen I, which was calcified at the end of 12 days.

RT‐PCR analysis showed that PAC1R (310 bp) was clearly expressed in hADSCs (Fig. 2D). Western blot analysis indicated that hADSCs expressed PAC1R (53 kD; Fig. 2E). Immunofluorescence assays also showed PAC1R expression in hADSCs (Fig. 2F). These assays demonstrated that PAC1R mRNA and protein were expressed in hADSCs.

### The effects of maxadilan on hADSC growth and migration

**Figure 3:**
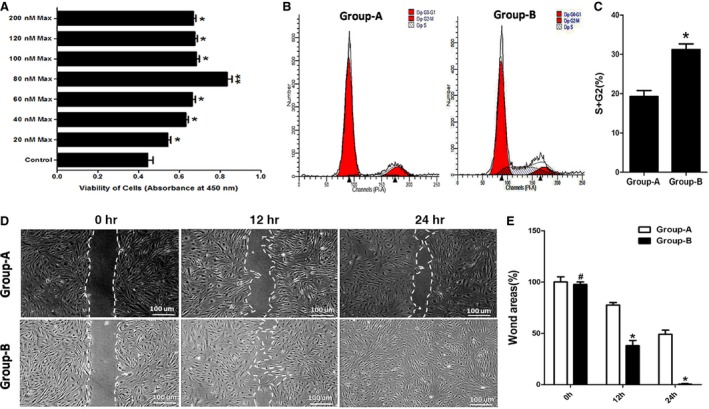
The effects of maxadilan on hADSC growth and migration. (**A**) The proliferation of hADSCs treated with maxadilan (0 nM (Control), 20, 40, 60, 80, 100, 120 and 200 nM) was detected using CCK‐8 assays. (**B**) The proliferation of hADSCs in group‐A and group‐B was analysed using cell cycle assays. (**C**) Quantification of the cell cycle assays. (**D**) Wound‐healing assays of hADSCs in group‐A and group‐B. (**E**) Quantification of the wound‐healing assays. Differences with **P < 0.01 (80 nM Max *versus* Control), *P < 0.05 (20, 40, 60, 100, 120 or 200 nM Max *versus* Control) or (group‐B *versus*. group‐A) were considered significant. Differences with ^#^ *P* > 0.05 (group‐B *versus*. group‐A) were considered not significant.

Compared with the control (0 nM), maxadilan (20, 40, 60, 80, 100, 120 and 200 nM) could promote hADSC proliferation according to CCK‐8 assays (*P < 0.05). The optimal concentration of maxadilan was found to be 80 nM (**P < 0.01; Fig. 3A). Human adipose‐derived stem cell proliferation was enhanced by 80 nM maxadilan (group‐B) compared with hADSCs that were not exposed to maxadilan (group‐A), as determined in cell cycle assays (Fig. 3B). The percentages of hADSCs entering the S and G2 phases in group‐A were 19.81 ± 1.44%, and group‐B was 31.65 ± 1.53% (Fig. 3C). The proliferative cells in group‐B were more 11.84 ± 1.22% than those in group‐A (*P < 0.05). These assays revealed that maxadilan could enhance hADSC proliferation.

The effects of maxadilan on hADSC migration were analysed using wound‐healing assays. At 0 hr, hADSCs in group‐A almost had the same wound area with group‐B (^#^ *P* > 0.05). After 12 hrs, there was a 22.54% decrease in the wound area compared with 0 hr in group‐A, while the wound area decreased by 59.52% in group‐B (*P < 0.05). At 24 hrs, the wound area of group‐A decreased by 51.02%, but the wound area in group‐B was almost closed (*P < 0.05; Fig. 3D). Statistical analysis of the wound areas over time according to ImageJ software revealed significantly lower wound areas in group‐B compared with group‐A at 12 and 24 hrs (Fig. 3E), which suggested that maxadilan could improve hADSC migration.

### The anti‐apoptotic effects of maxadilan on hADSCs

**Figure 4:**
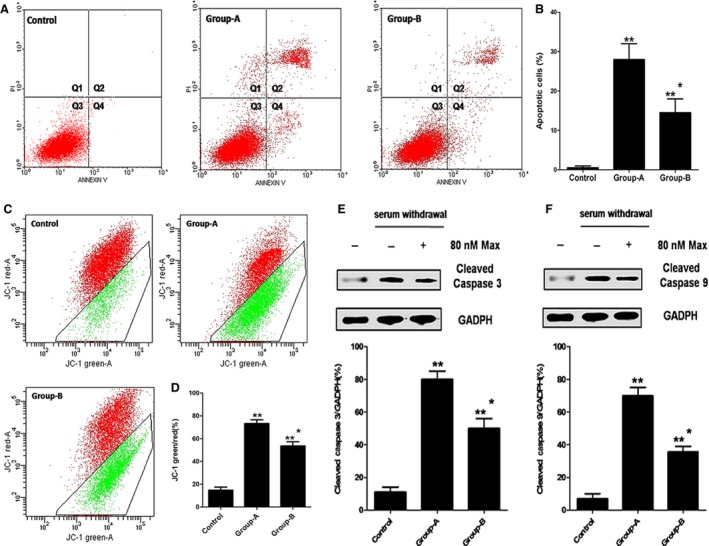
The anti‐apoptotic effects of maxadilan on hADSCs after serum withdrawal. (**A**) Annexin V and PI assays performed to detect the apoptotic of hADSCs in control, group‐A and group‐B by flow cytometry. Q1 area represents cell necrosis; Q2 is late‐apoptotic cells; Q3 is viable cells; Q4 is early‐apoptotic cells. (**B**) Quantification of the Annexin V and PI assays. (**C**) Mitochondrial membrane potential assays performed to assess the apoptotic of hADSCs in these groups. **(D**) Quantification of mitochondrial membrane potential assays. (**E**) Assessment of the expression of Cleaved Caspase 3 of hADSCs in these groups using Western blot assays. (**F**) Detection of the expression of Cleaved Caspase 9 of hADSCs in these groups using Western blot assays. Differences with **P < 0.01 (group‐A or group‐B *versus* control), *P < 0.05 (group‐B *versus* group‐A) were considered significant.

Human adipose‐derived stem cells in control were cultured in medium without 80 nM maxadilan and serum withdrawal treatments. During the early stage of apoptosis, cell typically has an intact cell membrane that is not stained with PI. However, externalization of phosphatidylserine (membrane phospholipids) can be detected by Annexin V. The percentage of apoptotic cells was calculated from the Q2 and Q4 areas. Annexin V and PI assays showed that maxadilan could decrease the ratio of apoptosis in hADSCs with serum withdrawal treatment (Fig. 4A). Control had just 0.55 ± 0.01% apoptosis cells. Human adipose‐derived stem cells in group‐A with serum withdrawal treatment demonstrated 28.32 ± 1.12% apoptotic cells, whereas the hADSCs in group‐B with serum withdrawal treatment demonstrated 14.53 ± 1.35% apoptotic cells. The apoptotic cells in both group‐A and group‐B were much more than control (**P < 0.01). There were 13.79 ± 1.03% increases of apoptotic cells in group‐A compared with group‐B (*P < 0.05; Fig. 4B).

The loss of mitochondrial membrane potential (▵Ψ m) was associated with the apoptosis. The ▵Ψ m in apoptosis cells was lower than that in living cells. JC‐1 was the probe which could enter the mitochondria. When the ▵Ψ m was low, it would form the monomer and glow green fluorescence. When the ▵Ψ m was high, JC‐1 would form polymer and glow red fluorescent. The ratio of JC‐1‐green/red could reflect the result of the apoptosis of cells. A high ratio of JC‐1‐green/red indicates a higher level of apoptosis than a low ratio. The mitochondrial membrane potential assays showed that maxadilan could reduce the ratio of JC‐1‐green/red in hADSCs with serum withdrawal treatment (Fig. 4C). The ratio of JC‐1‐green/red in control was 17.50 ± 1.34%. hADSCs in group‐A after serum withdrawal had a JC‐1‐green/red ratio of 76.58 ± 2.12%, whereas the hADSCs in group‐B after serum withdrawal had a ratio of 57.21 ± 1.94%. The ratio of JC‐1‐green/red in group‐A or group‐B was much more than control (**P < 0.01), and group‐A was more 19.37 ± 0.18% than group‐B (*P < 0.05; Fig. 4D).

When cells went into apoptosis, the Caspase 3 and Caspase 9 would be activated into Cleaved Caspase 3 and Caspase 9. Western blot assays showed that maxadilan could reduce the generation of Cleaved Caspase 3 and Caspase 9 in hADSCs with serum withdrawal treatment. The expression levels of Cleaved Caspase 3 and Caspase 9 in both group‐A and group‐B after serum withdrawal were higher than control (**P < 0.01), and hADSCs in group‐B exhibited 31% and 35.5% decreases in the expression levels of Cleaved Caspase 3 and Caspase 9, respectively, compared with those in group‐A (*P < 0.05; Fig. 4E and F). These results demonstrated that maxadilan could reduce hADSC apoptosis after serum withdrawal.

### The effects of maxadilan on the chemical neural induction of hADSCs

**Figure 5:**
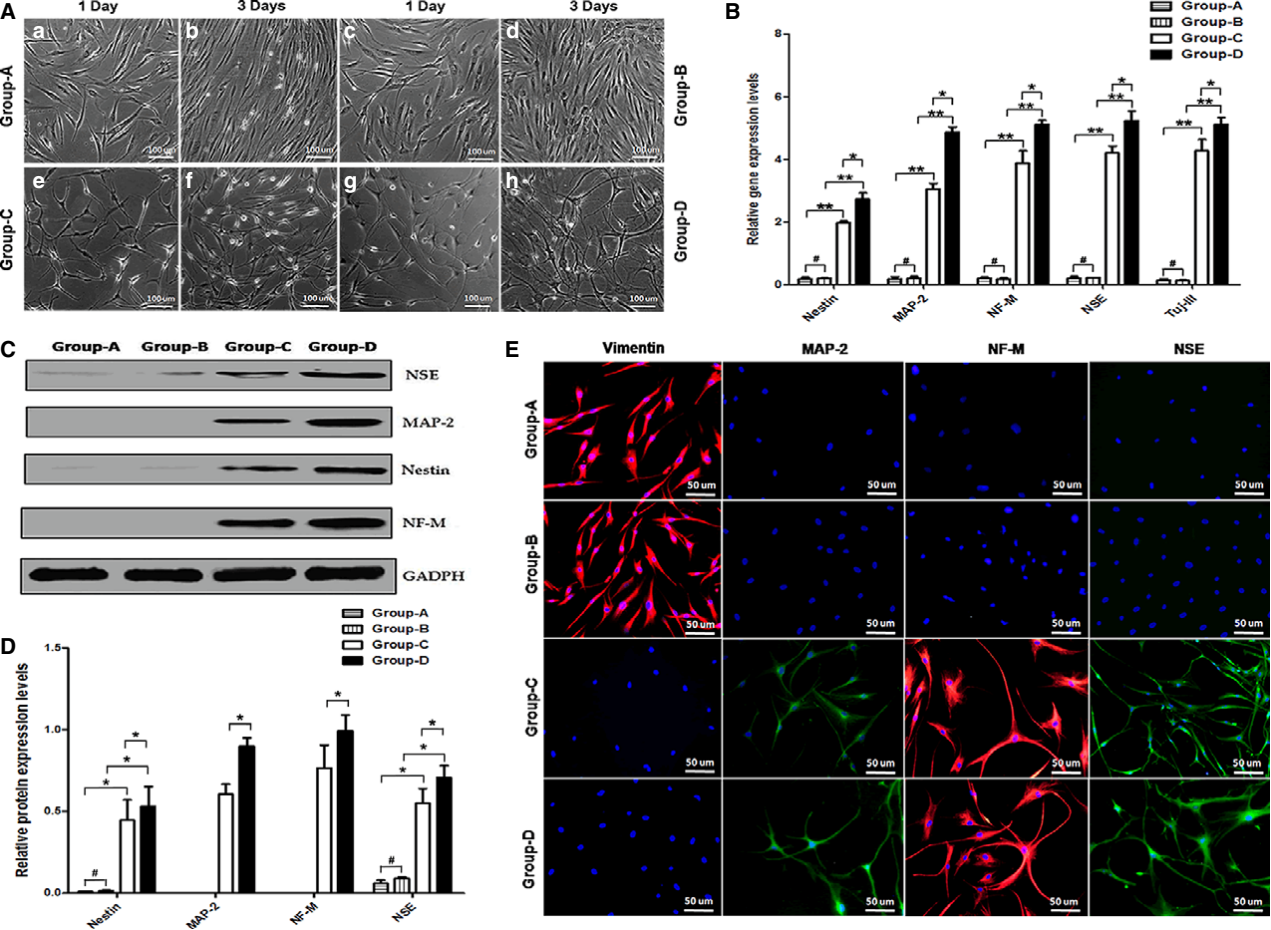
The effects of maxadilan on the chemical neural induction of hADSCs. (**A**) hADSCs treated without or with maxadilan were induced in chemical neural induction medium (group‐C and group‐D) or in hADSC culture medium (group‐A and group‐B) for 1 or 3 days. (**B**) Comparison of the relative gene expression levels of Nestin, MAP‐2, NF‐M, NSE and Tuj‐III in different groups on day 3 using qPCR analysis. (**C**) Assessment of protein expressions of NSE, MAP‐2, Nestin and NF‐M in different groups on day 3 using Western blot assays. (**D**) Quantification of protein expression levels of Western blot. (**E**) Detection of protein expressions of Vimentin, MAP‐2, NF‐M and NSE of hADSCs in different groups on day 3 using immunofluorescence assays. Differences with *P < 0.05 and **P < 0.01 were considered significant. Differences with ^#^ *P* > 0.05 were considered not significant.

In hADSC culture medium, hADSCs at P2 treated without maxadilan (group‐A) or with 80 nM maxadilan (group‐B) exhibited fibroblastic spindle‐shaped morphologies (Fig. 5Aa, Ab, Ac and Ad). However, after 1 day of chemical neural induction, the partial hADSCs (group‐C) demonstrated cell cytoplasm retraction towards the nucleus and the formation of contracted cell bodies with extended cytoplasmic extensions. Human adipose‐derived stem cells exhibited progressively robust neurite extension and neuronal morphology (Fig. 5Ae). Human adipose‐derived stem cells on the third day of chemical neural induction revealed a more developed neuron‐like morphology with long and branched cytoplasmic processes (Fig. 5Af). Our findings were consistent with previous reports 44, 45. Human adipose‐derived stem cells exposed to maxadilan treatment in chemical neural induction medium (group‐D) partially differentiated into neuronal‐like cells on day 1 (Fig. 5Ag), and the majority were differentiated into neural‐like cells and crossed with each other on day 3 (Fig. 5Ah).

The qPCR results showed that hADSCs after 3 days of chemical neural induction could significantly express marker genes of neural cells (Nestin, MAP‐2, NF‐M, NSE and Tuj‐III). However, hADSCs in hADSC culture medium scarcely expressed these genes. Importantly, in neural induction medium, maxadilan could up‐regulate the expression levels of neural marker genes (Fig. 5B). Western blot results revealed that hADSCs in both group‐C and group‐D could express the marker proteins NSE, MAP‐2, Nestin and NF‐M, however, hADSCs in groups‐A and group‐B hardly expressed MAP‐2 and NF‐M proteins and exhibited little expression of the NSE and Nestin proteins (Fig. 5C). In particular, in chemical neural induction medium, maxadilan also could up‐regulate the neural marker protein expression levels of NSE, MAP‐2, Nestin and NF‐M (Fig. 5D). These data suggested that maxadilan could improve the potential of hADSC differentiation into neural‐like cells in chemical neural induction medium.

In addition, immunofluorescence assays also were performed in hADSCs of group‐A and group‐B before chemical neural induction, and the cells demonstrated positive expression of Vimentin, but negative expression of the neural biomarkers MAP‐2, NF‐M and NSE. After chemical neural induction for 3 days, hADSCs treated without maxadilan (group‐C) or with 80 nM maxadilan (group‐D) exhibited negative expression of Vimentin but positive staining of the neural biomarkers MAP‐2, NF‐M and NSE (Fig. 5E).

### The effect of maxadilan on morphology of hADSCs in chemical neural induction medium

**Figure 6:**
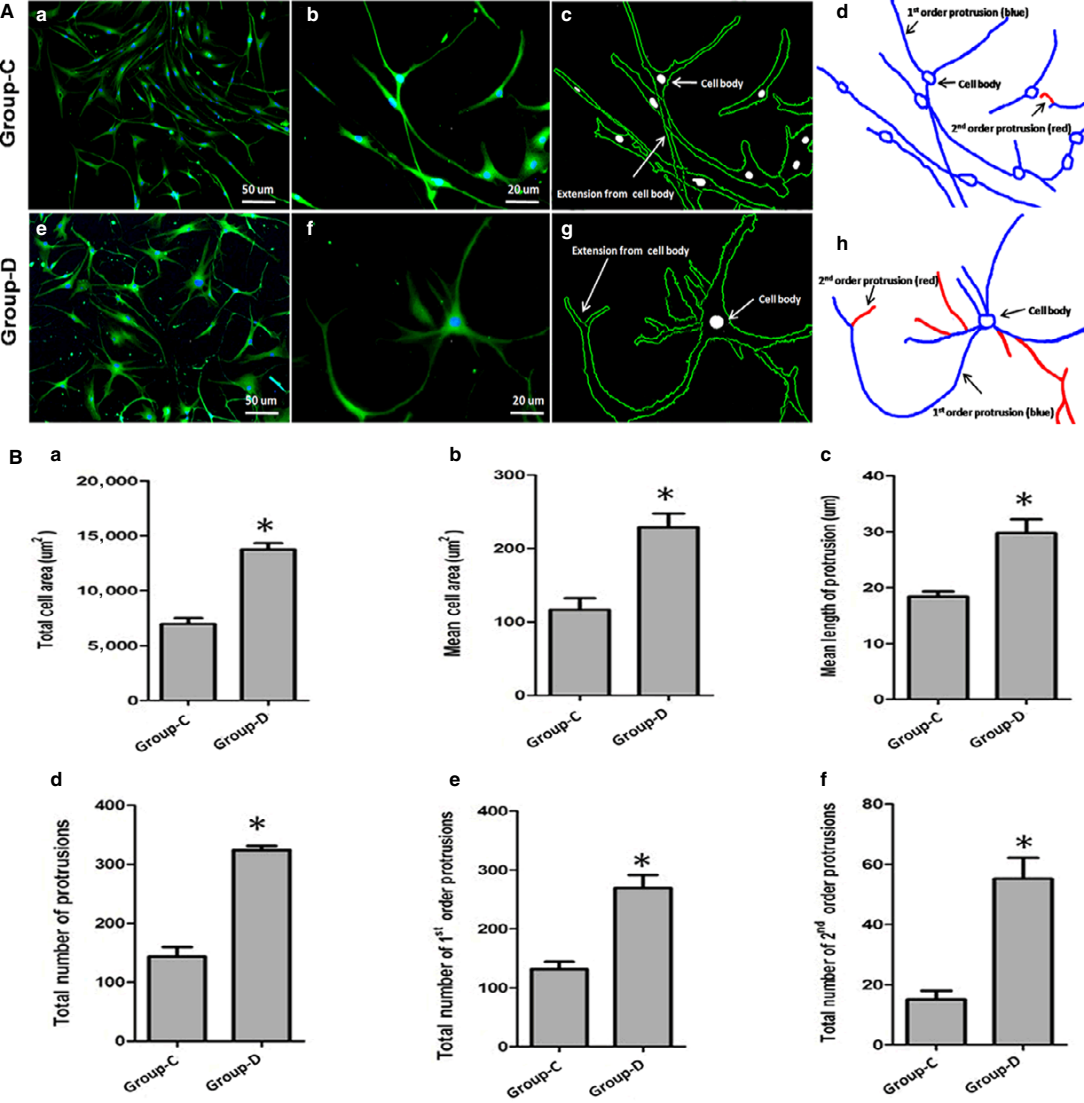
Morphological analysis of the effects of maxadilan on the chemical neural induction of hADSCs. (**Aa**,** Ab**,** Ae**,** Af**) Fluorescent images indicating cell contour in group‐C and group‐D. (**Ac**,** Ad**,** Ag**,** Ah**) The cell morphology was acquired from fluorescent images by MATLAB software, which identified the cellular contour (**Ac** and **Ag**) and cellular skeleton (**Ad** and **Ah**) in both groups. (**B**) Based on the cellular contour and skeleton, the dependence of cell morphologies was examined, including total cell areas (**Ba**), mean cell areas (**Bb**), the mean protrusion length (**Bc**), the total number of protrusions (**Bd**), 1st order protrusion numbers (**Be**), and 2nd order protrusion numbers (**Bf**) in two groups. Differences with *P < 0.05 (group‐C *versus* group‐D) were considered significant.

NSE immunofluorescence was assessed in cells in group‐C (Fig. 6Aa and Ab) and group‐D (Fig. 6Ae and Af) after 3 days of chemical neural induction. Using a custom‐designed program written in MATLAB, the cellular contours were obtained from the fluorescent images to identify the cell body and protrusions in group‐C (Fig. 6Ac) and group‐D (Fig. 6Ag). Then, based on the cellular contours, the morphological skeletons were determined, and the protrusions were classified into 1st order protrusions and 2nd order protrusions depending on their starting position (Fig. 6Ad and Ah). In addition, cell morphologies were carefully quantified with MATLAB software (Fig. 6B), including total cell areas, mean cell area, mean length of protrusions, total number of protrusions, the number of 1st order protrusions, and the number of 2nd order protrusions. The cell sizes were determined based on the area of the cellular contours, including the cell bodies and protrusions. The sizes of hADSCs in group‐D were higher than those in group‐C (*P < 0.05; Fig. 6Ba and Bb), which was related to the increased lengths and number of protrusions (*P < 0.05; Fig. 6Bc and Bd). The area per cell of group‐D was almost twice that of group‐C (*P < 0.05; Fig. 6Bb), and the protrusion length per cell increased when supplemented with maxadilan (*P < 0.05; Fig. 6Bc). The total number of protrusions in the cells of group‐D was almost twice that of group‐C (*P < 0.05; Fig. 6Bd). Interestingly, the total number of 1st order protrusions in group‐D was almost more twice than group‐C (*P < 0.05; Fig. 6Be). The total number of 2nd order protrusions in group‐D was also more than that in group‐C (*P < 0.05), but both were very few (Fig. 6Bf). Significantly, in chemical neural induction medium, maxadilan induced an efficient differentiation of hADSCs into neural‐like cell morphology.

### The effects of maxadilan on the cytokine induced neural redifferentiation of hADSCs

**Figure 7:**
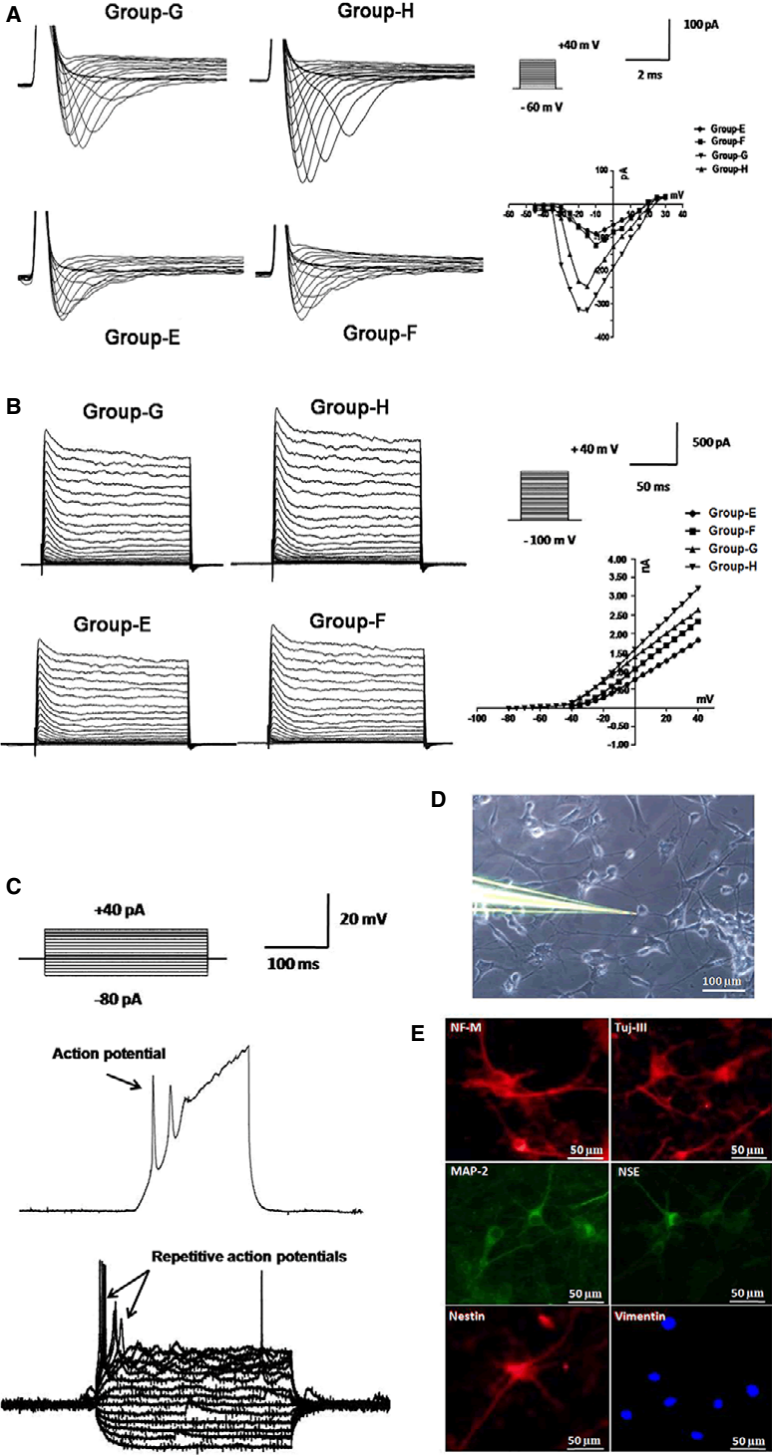
Electrophysiological analyses of cytokine neural redifferentiated hADSCs in different groups. (**A**) The holding potential was −60 mV and depolarizing steps were ranged from −60 to +40 mV at pulse of 2 msec. Representative traces of sodium currents and I–V curves demonstrated the voltage dependence of sodium currents in different groups (*n* = 20). (**B**) Sodium currents were blocked using 1 μM TTX and voltage‐dependent sodium currents were activated from a depolarizing step in mV intervals from −100 to +40 mV at pulse of 50 msec. Representative traces of potassium currents and I–V curves demonstrated the voltage dependence of sodium currents in different groups (*n* = 25). (**C**) Step current injection protocols were used from −80 to +40 pA at pulse of 100 msec. Representative traces showed repetitive action potentials in group‐H (*n* = 6) after cytokine neural redifferentiation for 2 weeks. (**D**) Typical recording of single whole‐cell patch‐clamp mode. (**E**) Detection of protein expressions of NF‐M, Tuj‐III, MAP‐2, NSE, Nestin and Vimentin in group‐H using immunofluorescence assays.

Electrophysiological current recordings could not be performed on chemical neural differentiated hADSCs because the cells were not easily approached by the patch‐pipette. Furthermore, the report previously demonstrated that dedifferentiated MSCs exhibited enhanced higher efficacy in neuronal redifferentiation compared to non‐manipulated MSCs 46. Dedifferentiated muscle stem cells could form neurosphere‐like structures that contained neural stem/progenitor cells 47. After cytokine neural redifferentiation *via* neurotrophic factors for 2 weeks, the cells could be more easily sealed for membrane current analysis using a whole‐cell patch clamp (Fig. 7D). More than sixty per cent of cells in group‐E, group‐F, group‐G and group‐H expressed prominent voltage‐dependent sodium currents and potassium currents. Sodium current recordings were detected during cell step depolarization within the physiological range from −60 to +40 mV at pulse of 2 msec. The mean peak amplitude of voltage‐dependent sodium currents was 100 ± 36 pA, while the peak sodium currents were 98 ± 6 pA (group‐E, *n* = 20), 121 ± 8 pA (group‐F, *n* = 20), 249 ± 11 pA (group‐G, *n* = 20) and 318 ± 12 pA (group‐H, *n* = 20), indicating the activation of sodium voltage‐gated channels, and group‐H was the highest (Fig. 7A). Along with sodium current induction, the cells demonstrated sustained outward currents that exhibited voltage dependence and kinetic characteristics for delayed rectifier potassium currents. When sodium currents were blocked using 1 μM TTX, we performed electrophysiological studies to investigate outward potassium currents. Potassium current recordings were examined during cell step depolarization within the physiological range from −100 to +40 mV at pulse of 50 msec. The mean peak amplitude of voltage‐dependent potassium currents was 500 ± 23 pA. Under current clamp conditions, the peak potassium currents were 1821 ± 68 pA (group‐E, *n* = 25), 2452 ± 87 pA (group‐F, *n* = 25), 2512 ± 79 pA (group‐G, *n* = 25) and 3433 ± 121 pA (group‐H, *n* = 25), indicating the activation of potassium voltage‐gated channels, and group‐H was the highest (Fig. 7B). Cells were maintained at a resting membrane potential and step current injection protocols were used from −80 to +40 pA at pulse of 100 msec. A few cells (*n* = 6) in group‐H demonstrated repetitive action potentials, indicating that these cells had the functional characteristics of neurons, but none of the cells in the other groups demonstrated repetitive action potentials (Fig. 7C). In addition, immunofluorescence assays indicated negative expression for Vimentin but positive staining of NF‐M, Tuj‐III, MAP‐2, NSE and Nestin in group‐H (Fig. 7E). These data demonstrated maxadilan could further strengthen cytokine neural redifferentiation into functional neurons in hADSCs.

### The potential molecular mechanisms underlying the promotion of hADSC viability and neural differentiation potential effects induced by maxadilan

**Figure 8:**
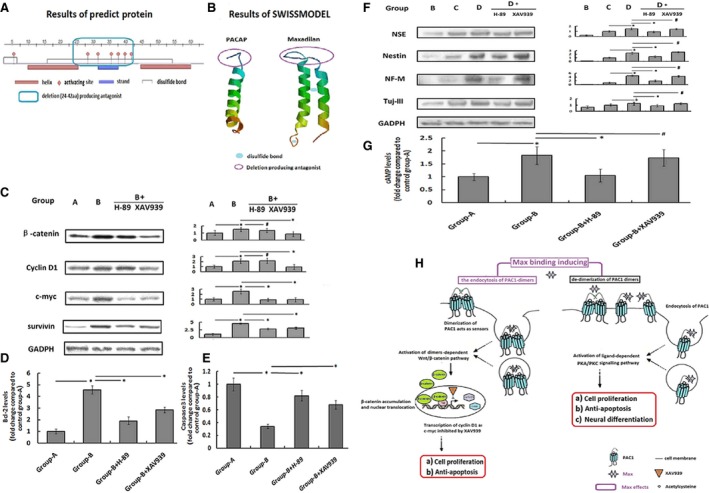
The potential molecular mechanisms underlying the promotion of hADSC viability and neural differentiation potential effects induced by maxadilan. (**A**) Domain structure analysis of maxadilan using PredictProtein. (**B**) The 3D structures of PACAP and maxadilan using SWISSMODEL. (**C**) Assessment of protein expression levels of β‐catenin, Cyclin D1, c‐myc and survivin in different groups using Western blot assays. (**D**) Detection of the Bcl‐2 levels in different groups using ELISA. (**E**) Comparison of the Caspase 3 activity in different groups using Caspase 3 activity assays. (**F**) Assessment of protein expression levels of NSE, Nestin, NF‐M and Tuj‐III in different groups using Western blot assays. (**G**) Quantification of the cAMP levels in different groups using ELISA. (**H**) Diagram of the potential molecular mechanisms underlying the promotion of hADSC viability and neural differentiation potential effects induced by maxadilan. Differences with *P < 0.05 were considered significant. Differences with ^#^ *P* > 0.05 were considered not significant.

A structural study of maxadilan showed that maxadilan has two PACAP‐like helixes combined with two intra‐molecular disulphide bonds 28. The structure analysis results for the maxadilan structure in PredictProtein (Fig. 8A) and SWISSMODEL (Fig. 8B) indicated that maxadilan with two PACAP‐like helixes had a PACAP‐dimer‐like structure.

The Wnt/β‐catenin signalling pathway is the classic pathway controlling cell proliferation and apoptosis 26. The results of Western blot analysis showed that the levels of β‐catenin and its target proteins Cyclin D1, c‐myc and survivin in group‐B were higher than those in group‐A (*P < 0.05). However, all proteins could be down‐regulated by XAV939 (*P < 0.05), an inhibitor of the Wnt/β‐catenin signalling pathway (Fig. 8C). At the same time, the up‐regulated Bcl‐2 levels and the down‐regulated Caspase 3 activity in group‐B compared with group‐A after serum withdrawal were also inhibited by XAV939 (*P < 0.05; Fig. 8D and E). The results demonstrated that the Wnt/β‐catenin signalling pathway, which is associated with the dimer‐dependent activity of PAC1R 26, might be involved in the cell proliferative and anti‐apoptotic activities of maxadilan (Fig. 8Ha and Hb).

In addition, the PKA signalling pathway inhibitor H‐89 not only decreased the levels of c‐myc and survivin promoted by maxadilan compared with group‐B (*P < 0.05; Fig. 8C) but also inhibited the effects of maxadilan on intracellular Bcl‐2 levels and Caspase 3 activity (*P < 0.05; Fig. 8D and E). The results revealed that the PKA signalling pathway, which is associated with the ligand‐dependent activity of PAC1R 48, might be also involved in the cell proliferative and anti‐apoptotic activities of maxadilan (Fig. 8Ha and Hb).

For chemical neural differentiation of hADSCs, consistent with the cAMP assay results (*P < 0.05; Fig. 8G), maxadilan could increase the expression levels of neural markers including NSE, Nestin, NF‐M and Tuj‐III in group‐D compared with group‐C (*P < 0.05), and such effects could be inhibited by the PKA signal inhibitor H‐89 (*P < 0.05). However, the Wnt/β‐catenin signalling pathway inhibitor XAV939 did not interfere with the neural differentiation promoted by maxadilan (^#^ *P* > 0.05; Fig. 8F). The results indicated that the ligand‐dependent activity of PAC1R mediated by the PKA signalling pathway but not dimer‐dependent activity of PAC1R by the Wnt/β‐catenin signalling pathway might contribute to the effects of maxadilan on promoting neural differentiation (Fig. 8Hc).

## Discussion

Human adipose‐derived stem cells are a heterogeneous group of adult stem cells found in adipose tissues. In recent years, there had been many studies indicated that hADSCs had potentiality for clinical application. In our study, we reported an efficient method for obtaining primary hADSCs from abdomen and thigh through liposuction. The surface antigens and morphology of harvested hADSCs were consistent with reported articles 49, 50.

PAC1R, a PACAP‐specific receptor, is expressed in embryonic stem cells, and the expression of PAC1R mRNA is further up‐regulated after differentiation into neurons 51, 52. Recently, we reported that PAC1R is also present in human induced pluripotent stem cells 53. However, nothing is known regarding the presence of PAC1R in hADSCs. And we first demonstrated PAC1R expression in hADSCs by gene testing and protein assay.

In this study, it was revealed that maxadilan, a specific PAC1R agonist 28, 29, 30, 31, 54, could increase hADSC proliferation and promoted migration. It could also counteract hADSC apoptosis. Human adipose‐derived stem cells after serum withdrawal exhibited the down‐regulation of Cleaved Caspase 3 and Caspase 9 as well as Bcl‐2 up‐regulation when supplemented with maxadilan. It is known that Caspase plays a key role during the effect and initiation phase of apoptotic cell death. Caspase 3 and Caspase 9 are prototypical caspase and are important for apoptosis execution 55. Bcl‐2 is an anti‐apoptotic protein in the Bcl‐2 family 56.

Many previous studies reporting the generation of neurons from ADSCs or MSCs were based on the morphological identification and expression of neuronal markers, such as MAP‐2, NF‐M, Tuj‐III, NSE and Nestin 57, 58, 59, 60, 61. In our study, before chemical neural induction, hADSCs treated with or without maxadilan were found almost negative expression of the neural biomarkers MAP‐2, NF‐M, Tuj‐III, NSE and Nestin. But after chemical neural induction for 3 days, hADSCs treated with or without maxadilan exhibited positive staining of the neural biomarkers MAP‐2, NF‐M, Tuj‐III, NSE and Nestin. These data confirm that the neural differentiation of hADSCs was mainly medium‐dependent but not maxadilan‐dependent. However, we observed that maxadilan indeed enhanced hADSC neural differentiation potential. Using MATLAB software analysis, we found that cell sizes, protrusion numbers, and protrusion lengths were increased when hADSCs were treated with maxadilan in chemical neural induction medium. The qPCR and Western blot results confirmed that maxadilan could promote hADSCs to more effectively differentiate into neural‐like cells in neural induction. Moreover, we showed for the first time that maxadilan could further strengthen cytokine neural redifferentiation into functional neurons in hADSCs. Function test of electrophysiology showed that the 60% of cells had prominent voltage‐dependent sodium currents and potassium currents after cytokine neural redifferentiation *via* neurotrophic factors in hADSCs for 2 weeks. The peak sodium or potassium currents of the cells with maxadilan were higher. The repetitive action potentials were also present in a few of these redifferentiated cells only with maxadilan. Mercer *et al*. revealed that maxadilan stimulate adult mouse neural stem cell proliferation through PAC1R activation 62. Nishimoto *et al*. discovered that maxadilan could increase the number of BrdU/Nestin immunoreactive neural progenitor cells with the elongation of cell processes possessing stellate and astrocyte‐like morphologies. Maxadilan induced marked increase in GFAP immunoreactive astrocytes 63. PAC1R could stimulate the neuritogenesis by increasing both the number and length of processes 64, 65. Our results were consistent with previous reports and further discovered that maxadilan had a promoting role in neural differentiation and redifferentiation.

Pituitary adenylate cyclase‐activating polypeptide (11‐38) with an α‐helix structure is a cell‐penetrating peptide that induces dimer‐dependent activity of the linked molecule 66. PAC1R, a PACAP‐specific receptor, possesses both dimer‐dependent activity associated with the Wnt/β‐catenin signalling pathway 26 and ligand‐dependent activity through the PKA signalling pathway 67. The structure of maxadilan, containing two α‐helix structures, is similar to PACAP (Fig. 8B). Furthermore, maxadilan mediated effects that were different from PACAP after the activation of PAC1R 30, which suggested that the PACAP‐dimer‐like structure of maxadilan may effectively bind to the dimer of PAC1R and generate biological activities that are different from PACAP. The α‐helix structure of PACAP, which has amphiphilic (both hydrophilic and lipophilic) properties, has been shown to enter into cells *via* dimer‐dependent cellular uptake 68. The structural analysis showed that maxadilan with a PACAP‐dimer‐like structure had two groups of α‐helix structures with amphiphilic properties. In theory, maxadilan could effectively induce the internalization of PAC1R after binding to PAC1R.

The Wnt/β‐catenin signalling pathway is the classic pathway controlling cell proliferation and apoptosis 26. A closely related report demonstrated that XAV939 (an inhibitor of the Wnt/β‐catenin signalling pathway) promoted cell apoptosis in the neuroblastoma cell line SH‐SY5Y 69. A previous study showed that PAC1R had intrinsic activity associated with the internalization of the PAC1R‐dimer and mediated by the Wnt/β‐catenin signalling pathway, which rendered cells expressing PAC1R resistant to serum withdrawal‐induced apoptosis 26. In this study, it was discovered that maxadilan could up‐regulate the levels of β‐catenin in hADSCs expressing PAC1R and Cyclin D1 as well as c‐myc, which were the target proteins of the Wnt/β‐catenin signalling pathway. The effects of maxadilan on β‐catenin and Cyclin D1 were inhibited only by XAV‐933 but not by the PKA signalling pathway inhibitor H‐89. These data indicated that maxadilan might enhance the intrinsic activity of PAC1R through binding to the PAC1R dimer and inducing its internalization, and then effectively promoted proliferation and inhibited apoptosis by up‐regulating the Wnt/β‐catenin signalling pathway. Meanwhile, maxadilan also increased the levels of c‐myc and survivin, which were inhibited by XAV939 and H‐89 showing that not only the dimer‐dependent activity but also the ligand‐dependent activity of PAC1R enhanced by maxadilan contributed to the proliferation and anti‐apoptosis effectors in hADSCs. XAV939 effectively suspended the pro‐proliferative and anti‐apoptotic effects of maxadilan, but it could not inhibit neural differentiation. Maxadilan effectively up‐regulated markers of neural differentiation such as NSE, Nestin, NF‐M and Tuj‐III, and could be specially inhibited by the PKA signalling pathway inhibitor H‐89. These results suggested that maxadilan might also act as an agonist of PAC1R to activate the ligand‐dependent activity of PAC1R by the classical PKA signalling pathway, and subsequently improved neural differentiation 70. cAMP‐PKA pathway was reported to be involved in induce neuronal differentiation, neurite outgrowth, neural survival, neuronal integrin function, long‐term memory and neural plasticity 61. Our cAMP assay results also suggested that it was PKA signalling pathway that contributed to the effects of maxadilan on the promotion of neural differentiation. Taken as a whole, our study discovered that both the dimer‐dependent activity of PAC1R mediated by the Wnt/β‐catenin signalling pathway and the ligand‐dependent activity of PAC1R mediated by the PKA signalling pathway might contribute to the proliferative and anti‐apoptotic activities by maxadilan. However, only PKA signalling pathway might be responsible for the positive effects of maxadilan on the neural differentiation of hADSC (Fig. 8H). While this study just hints the potential molecular mechanisms underlying the promotion of hADSC viability and neural differentiation potential effects induced by maxadilan, the convincing molecular mechanisms should need further study in the future.

In summary, we demonstrated that PAC1R was present in hADSCs. Maxadilan, a PAC1R‐specific agonist, could promote the neural differentiation and redifferentiation potential of hADSCs in neural differentiation medium. In addition, maxadilan enhanced hADSC proliferation and reduced hADSC apoptosis. The anti‐apoptotic role of maxadilan correlated with the down‐regulation of Cleaved Caspase 3 and Caspase 9 as well as up‐regulation of Bcl‐2. Both of Wnt/β‐catenin and PKA signalling pathways involved in hADSC proliferation and anti‐apoptosis after maxadilan treatment, while only the PKA signalling pathway accounted for neural differentiation.

## Conflicts of interest

The authors confirm that there are no conflicts of interest.
